# Machine learning for the development of new materials for a magnetic tunnel junction

**DOI:** 10.1038/s44306-025-00094-z

**Published:** 2025-07-30

**Authors:** Atsufumi Hirohata, Hiroki Koizumi, Tufan Roy, Masahito Tsujikawa, Shigemi Mizukami, Kenji Nawa, Masafumi Shirai

**Affiliations:** 1 Center for Science and Innovation in Spintronics, Tohoku University, Sendai, Japan; 2 Research Institute of Electrical Communication, Tohoku University, Sendai, Japan; 3 Max-Planck-Institute for Chemical Physics of Solids, Dresden, Germany; 4 WPI Advanced Institute for Materials Research, Tohoku University, Sendai, Japan; 5 Graduate School of Engineering, Mie University, Tsu, Japan; 6 Research Center for Magnetic and Spintronic Materials, National Institute for Materials Science (NIMS), Tsukuba, Japan; 7 National Institute of Advanced Industrial Science and Technology, Tsukuba, Japan; 8 Present Address: Research Institute of Electrical Communication, Tohoku University, Sendai, Japan

**Keywords:** Spintronics, Computational methods

## Abstract

In materials science, we have been increasing the number of constituent elements in an alloy and compounds to improve their properties. For example, in magnetism and spintronics, ternary alloys, such as NdFeB and CoFeB have been developed and widely used in permanent magnets and memories/sensors, respectively. It has now been considered to be a time to add more elements to further explore their horizon. For such a complicated development, a manual systematic study is no longer practical, leading to the utilisation of machine learning to predict a candidate. These candidates can then be additionally screened by ab initio calculations before experimental confirmation, which can be performed routinely. Additional use of quantum annealing may also broaden the adoptability of machine learning on the materials development. In this perspective, we plan to offer a standardised process for such a development with some requirements for improvement.

## Introduction

**Fig. 1:**
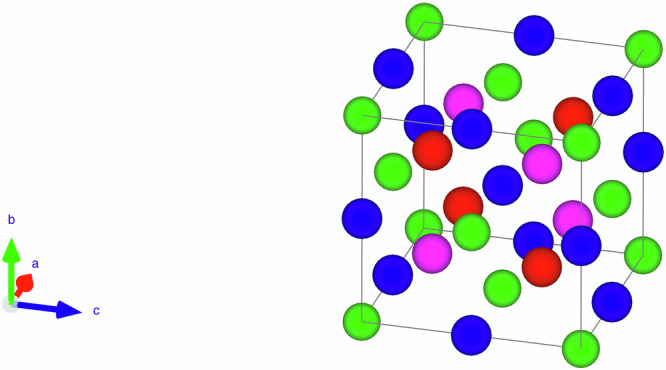
Schematic crystalline structure of a quaternary *XX’YZ* Heusler alloy (red, purple, blue and green atoms represent *X*, *X’*, *Y* and *Z* elements, respectively) created by VESTA [42].

In spintronics, one of the major methods to generate a spin-polarised electron is to flow an unpolarised electron through a ferromagnetic layer, e.g., giant magnetoresistive (GMR) and tunnelling magnetoresistive (TMR) junctions^1^. To date, the maximum GMR and TMR ratios have been reported to be 82^2^ and 631%^3^ at room temperature, respectively. In order to increase these ratios further at least over 1000% to achieve a magnetic switch, it is crucial to develop a half-metallic ferromagnetic film with 100% spin polarisation at room temperature^4,5^, with a suitable non-magnetic spacer and tunnel barrier for GMR and TMR devices. Among such half-metallic ferromagnets, a Heusler alloy can be an ideal candidate. Two distinctive types of Heusler alloys exist, half- and full-Heusler alloys with the form of *XYZ* and *X*_2_*YZ*, respectively. As shown in Fig. 1, these alloys have complicated crystalline structures, the *C*1_*b*_ (with making *X’* to be empty) and *L*2_1_ structure (with making *X’* to be the same as *X*), respectively. In order to develop an appropriate Heusler alloy for spintronic applications, some of *X* atoms can be replaced with *X’* to form a quaternary alloy to increase the alloy combinations as schematically shown in Fig. 1.

**Fig. 2:**
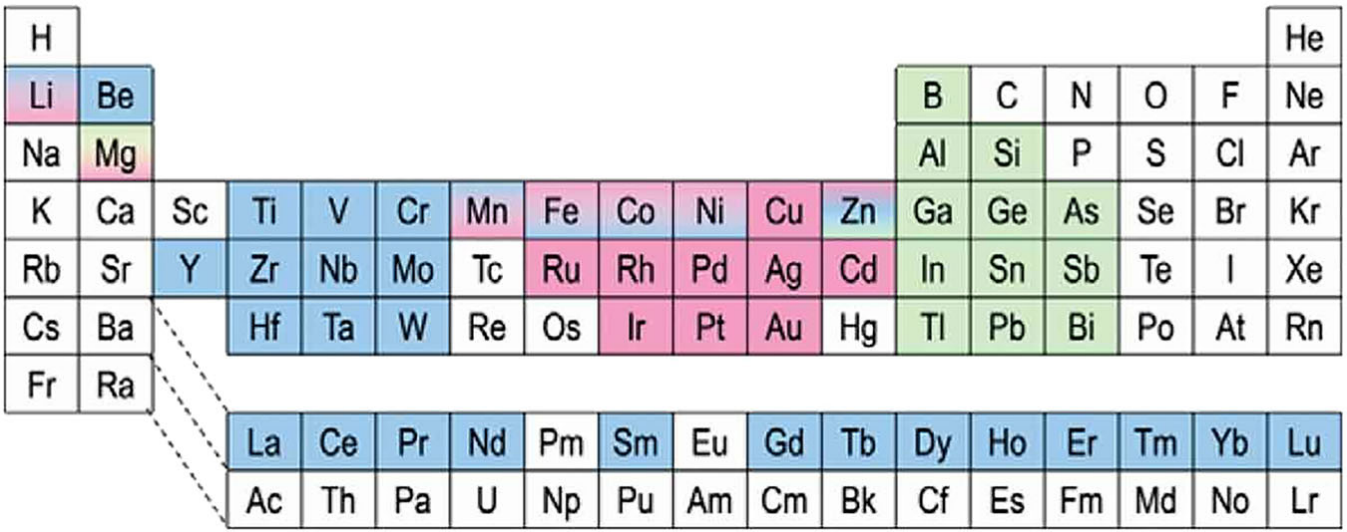
Consisting elements for quaternary Heusler alloy *XX’YZ*. On the periodic table, elements which can fill *X* and *X’*, *Y* and *Z* positions are highlighted in red, blue and green, respectively^5^.

For the Heusler alloys, 16, 30 and 14 elements can fill the *X*, *Y* and *Z* positions, respectively as listed in Fig. 2. This means there are 16 × {16 (for ternary and quaternary *L*2_1_) + 1 (vacancy for *C*1_*b*_)} × 30 × 14 = 114,240 combinations. It is hence impossible to study each alloy experimentally. Instead it is essential to utilise machine learning to make screening. This way one can efficiently predict an alloy even with minor compositional changes, which make the combinations to be infinite.

For the material development, there are nine major methods currently used; neural networks (NNs), support vector machine, linear regression, logistic regression, K-means clustering, K-nearest neighbours, decision trees, random forests and principal component analysis^6^. Each method has advantages and disadvantages. Note that irrelevant methods may provide wrong prediction. In order to make the selection of a method, a flowchart has been proposed by Okabe et al.^6^.

To date, the data-driven materials design, i.e., materials informatics, has been successfully applied for prediction of a large variety of spintronics materials, where the most attempts have been made in virtual space, i.e., only with machine learning and ab initio calculations^7–15^. For example, Kim et al. built a random forest approach combined with ab initio calculations to search for a quaternary Heusler alloy in a stable phase^7^. Hu et al. demonstrated predictions of Heusler alloys with high spin polarisation by fully connected NN and ab initio calculations^9^. Very recently, a stacked model approach, incorporating different machine learning models, has been proposed to be a robust technique and applied to predict novel Heusler alloys^12^. Bayesian optimisation is also widely used for transition-metal alloys^12,13^ and tunnel barrier materials^14^. An attempt to merge virtual space with such real space, i.e., machine learning and experiments, has been realised but with a limited number of examples, e.g., ref. ^15^.

**Fig. 3:**
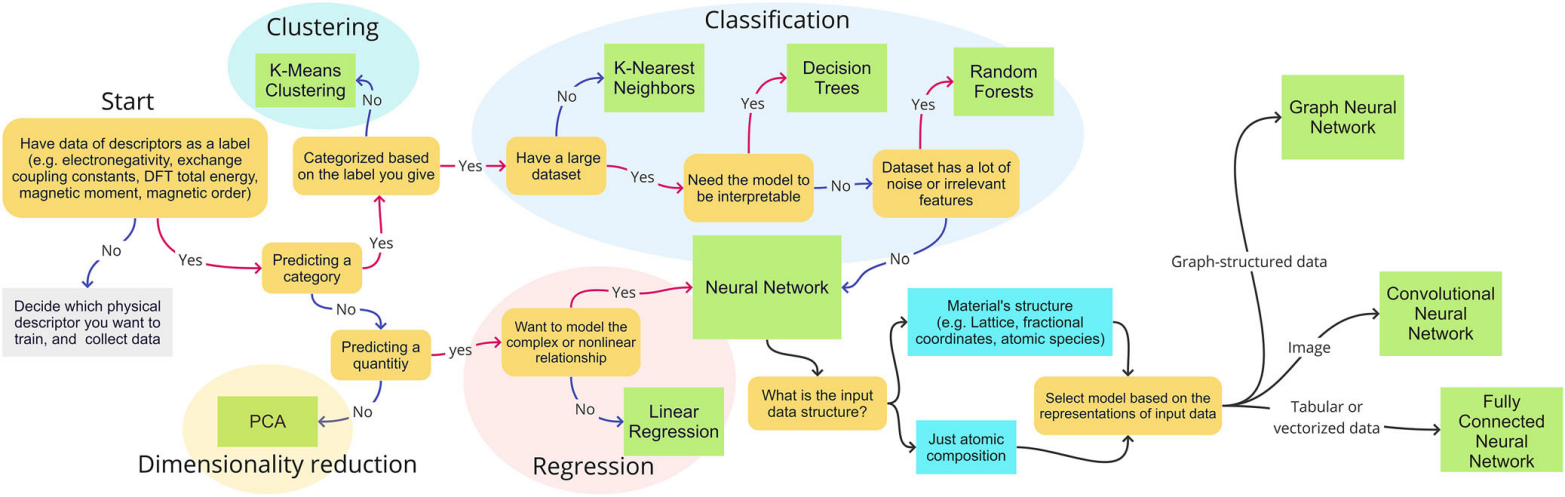
Flowchart for the selection of a relevant approach for machine learning^6^.

In this work, half-metal Heusler alloys suitable for spintronic device applications have been discovered by combining virtual-space prediction and real-space thin-film fabrication. For the prediction of a Heusler alloy, a decision tree has been selected according to the flowchart in Fig. 3. The decision tree method is relatively simple to use and interpret, and is to handle both numerical and categorical data with and without nonlinearity (see Section “Machine learning and first-principles Calculations”). However, the decision tree may create over-complexity if training data used are not representative. In addition, we have introduced the prediction of spinel-type oxides as a new tunnel barrier, where cation disordering determines thermodynamical stability and TMR properties as discussed in Section “Machine learning and first-principles Calculations for more complex materials”. Here, quantum annealing approach, a promising alternative method for a sufficient exploration of optimal materials from combinatorial explosion of candidates, is combined with machine learning and ab initio calculations.

## Machine learning and first-principles calculations

For the ab initio calculations, a projector augmented wave (PAW) method^16^ implemented in Vienna ab initio simulation package (VASP)^17^ was used with a plane wave basis set. A generalised gradient approximation (GGA)^18^ was adopted for exchange-correlation energies/potentials. The Liechtenstein’s method^19^ implemented in spin-polarised relativistic Korringa-Kohn-Rostoker (SPR-KKR)^20^ computational code was utilized to calculate an exchange interaction defined as

1 $$H=-{\sum }_{i\ne j}{J}_{ij}{\overrightarrow{{\boldsymbol{e}}}}_{i}\cdot {\overrightarrow{{\boldsymbol{e}}}}_{j}$$

where $\vec{{{\boldsymbol{e}}}_{i}}$ is a unit vector pointing to the direction of a magnetic moment at an atom $i$. In the framework of a mean-field approximation, Curie temperature (*T*_C_) was evaluated by solving the following linear equations in terms of the thermal average of a magnetic moment at a site $\mu$:

2 $$\langle {e}^{\mu }\rangle =\frac{2}{3{k}_{{\rm{B}}}{T}_{{\rm{C}}}}{\sum }_{\nu }{J}_{0}^{\mu \nu }\langle {e}^{\nu }\rangle$$

where ${J}_{0}^{\mu \nu }$ is the sum of exchange interactions between $\mu$ and $\nu$ sites up to the distance $R$ defined as^21^

3 $${J}_{0}^{\mu \nu }\equiv {\sum }_{R}{J}_{0{\rm{R}}}^{\mu \nu }$$

**Fig. 4:**
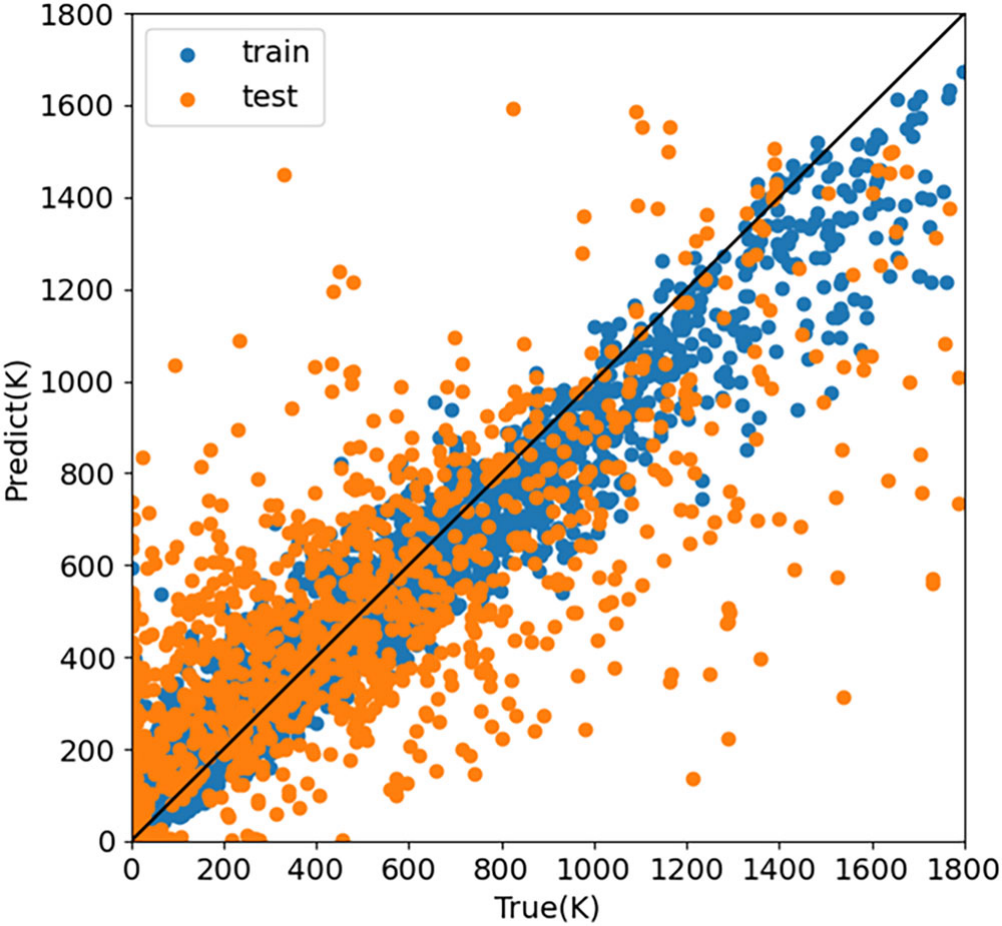
Regression analysis on *T*_C_ of quaternary Heusler alloys using Light GBM. Data used for training and predicted are shown as blue and amber dots, respectively.

For the Heusler-alloy prediction, regression analysis on *T*_C_ was carried out over the alloys consisting of *XX’YZ*. As a decision tree method, Light Gradient Boosting Machine (Light GBM) (https://lightgbm.readthedocs.io/en/stable/) was employed for the analysis. As shown in Fig. 4, about 4550 data were used for training, while about 1950 data were predicted as a preliminary test. Here, a correlation coefficient was calculated to be 0.91 and 0.73 for training and prediction, respectively.

Accordingly, 84 (178) Heusler alloys were preliminary predicted to possess *T*_C_ higher than 1000 K (800 K). Their *T*_C_ were then calculated by ab initio calculations and we found that 67 (132) Heusler alloys actually possess *T*_C_ higher than 1000 K (800 K), proving the accuracy of prediction to be about 80% (74%) for the criteria *T*_c_ > 1000 K (800 K). Note that additional 83 (104) Heusler alloys with their *T*_C_ higher than 1000 K (800 K), which were not predicted by the machine learning, were also included by ab initio calculations. After running full screening over about 97,000 alloys, the ML model predicted that additional 84 (362) Heusler alloys possess *T*_C_ higher than 1000 K (800 K). By using ab initio calculations, we confirmed that 62 (179) Heusler alloys actually possess *T*_C_ higher than 1000 K (800 K) among the predicted alloys. The accuracy of the ML prediction is evaluated as 74% (49%) for the full-range screening with the criteria *T*_C_ > 1000 K (800 K). Thus, the accuracy of the ML prediction is reasonable even in the full-range screening. Finally, we selected about 350 Heusler alloys for the candidates of electrode materials in magnetic tunnel junctions (MTJs) by taking account the *T*_C_ and also the stability of alloys based on the calculated formation energy. Note that a high *T*_*C*_, over 1000 K (800 K) ensures a stronger exchange coupling between the magnetic atoms of bulk electrode. This stronger coupling reduces the effect of thermal fluctuation of the magnetic moments, hence expected to reduce the temperature dependence of TMR ratio.

**Fig. 5:**
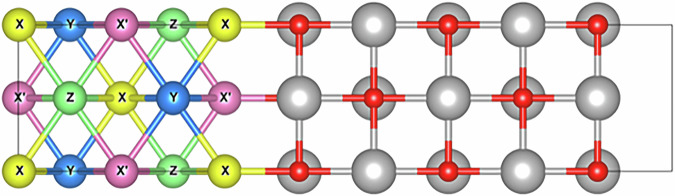
*XX’YZ*/MgO interface for calculations (*XX’*-termination) created by VESTA^42^.

For a realistic device application, magnetic stiffness was then calculated at an interface between the predicted Heusler-alloy film and a MgO tunnelling barrier (see Fig. 5). Including the abovementioned 350 candidates, the magnetic stiffness of about 600 *XX’YZ*/MgO junctions were assessed by ab initio calculations for both *XX’*- and *YZ*-terminated interfaces. As a result, we found the interfaces which possess relatively high magnetic stiffness as listed in Table 1. Note that the interface with higher magnetic stiffness is relevant for applications.

**Table 1:** Magnetic stiffness calculated at *XX’YZ*/MgO interfaces

Heusler alloy *XX’YZ*	Termination	Magnetic stiffness [mJ/m^2^]
Fe_2_CoAl (inverse)	CoFe/FeAl/MgO	616
Fe_2_CoGa (inverse)	CoFe/FeGa/MgO	581
Fe_2_CoIn (inverse)	CoFe/FeIn/MgO	530
Co_2_MnAl	CoCo/MnAl/MgO	497
CoCrMnIn	MnIn/CoCr/MgO	494
CoCrMnGa	MnGa/CoCr/MgO	485
Co_2_FeSi	FeSi/CoCo/MgO	480
CoCrMnSi	MnSi/CoCr/MgO	470

By comparing these stiffness constants calculated with known data, e.g., Fe/MgO: 753 mJ/m^2^ and Co_2_MnSi/MgO: 529 mJ/m^2^^22^, most interfaces can be implemented in a spintronic device possibly. In this perspective, CoCrMnSi and Fe_2_CoAl were selected for further experimental investigation. Here, strong antiferromagnetic coupling is formed between the constituent atoms, e.g., between Mn ( + 2.86 μ_B_) and Cr (–2.89 μ_B_) spins for CoCrMnSi, enhancing magnetic stiffness at the interface^23^.

Analysing the data calculated for *XX’YZ*/MgO junctions, we found the correlation between the magnetic stiffness constants on the *XX’YZ*/MgO interfaces and the magnitude of magnetic moment of *XX’YZ* averaged over the interfacial region. The covalent bonding with the nearest neighbouring O atom causes the reduction of magnetic moment at the magnetic atom on the interface, leading to the reduction of magnetic stiffness constants. The strength of the covalent bonding depends on the sorts of magnetic atoms on the interfacial layer and the atomic distances between the magnetic atoms and the neighbouring O atoms. Experimentally, one can expect that the formation of the energetically stable atomic termination needs advanced technique. For example, Yamada et al. tried to control the interface using molecular beam epitaxy (MBE) technique^24^. As various techniques are studied for interface control, thus we believe that it is important to further develop the growth technique to control the interface (please also see the review of the interface of Heusler alloys/compounds^25^).

To date, there is no experimental research focusing on the high-stiffness interface. One example of interface modification is that MTJs of Co_2_MnSi terminated with Co_2_MnAl at the interface. Miura et al. reported that an insertion of thin Co_2_MnAl layers into a Co_2_MnSi/MgO junction can eliminate interface states in the minority-spin gap and provide a 100% spin-polarisation at the interface^26^. Ozawa et al. reported a TMR ratio was enhanced by inserting a thin Co_2_MnAl layer at the Co_2_MnSi/MgO interface which was prepared by sputtering. MTJs fabricated by sputtering requires annealing to enhance TMR effect, which could also cause atomic mixing^27^. One strategy is to use a MBE method to form equilibrium/non-equilibrium state of the interface. For example, Brown-Heft et al. recently reported termination-dependent half-metallicity in Fe_2_MnAl/Co_2_MnAl superlattice prepared using MBE method^28^. Therefore, such MBE technique may be one of the promising technologies for translating simulations to real structures.

## Experimental investigation and discussion

Here we review two experimental studies trying to obtain the films of new Heusler alloys predicted from the abovementioned first-principes calculations, such as NiCrMnSi^29^ and CoIrMnAl^30^. All samples were deposited on MgO(001) substrates using an ultrahigh vacuum (UHV) magnetron sputtering.

**Fig. 6:**
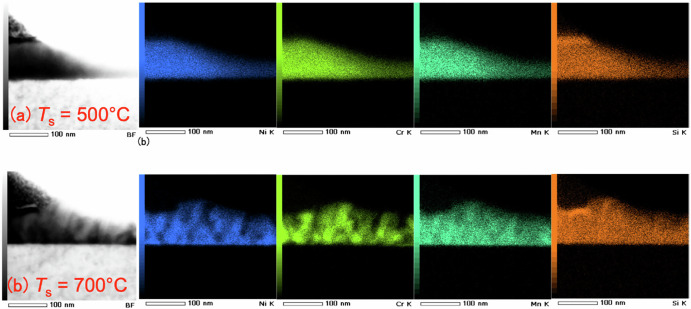
Structural analysis of NiCrMnSi films. Transmission electron microscopy images (left), and elements analysis (right) for NiCrMnSi films deposited at (**a**) 500 and (**b**) 700 °C. Blue, yellow green, and orange data points corresponding to the Ni, Cr, Mn and Si elements, respectively. Reprinted with permission from Onodera et al.^29^. © 2020 The Japan Society of Applied Physics.

Sample stacking structure of NiCrMnSi was MgO(001) substrate/NiCrMnSi(100 nm)/Ta(3 nm)^29^. NiCrMnSi films were deposited by a co-sputtering technique using NiCr and MnSi alloy targets. The composition of Ni : Cr : Mn : Si in the films was evaluated as 25.9 : 29.5 : 23.0 : 21.6 (at%). The out-of-plane x-ray diffraction (XRD) patterns showed the diffraction peaks in the films deposited at the temperature more than 700 °C. However, the XRD patterns were not consistent with those from the cubic or tetragonal phase of NiCrMnSi. Transmission electron microscopy (TEM) measurements indicated multiple crystalline phases were formed for NiCrMnSi films deposited at 700 °C. For the sample deposited at 500 °C, all four elements were homogeneously distributed in a whole region, indicated with energy dispersive X-ray spectrometry (EDX) in the TEM apparatus, as shown in Fig. 6. However, non-uniform distributions for Ni and Cr atoms were observed for the sample deposited at 700 °C. It suggested that the Ni-rich phase and the Cr-rich phase were spatially separated in this sample^29^.

**Fig. 7:**
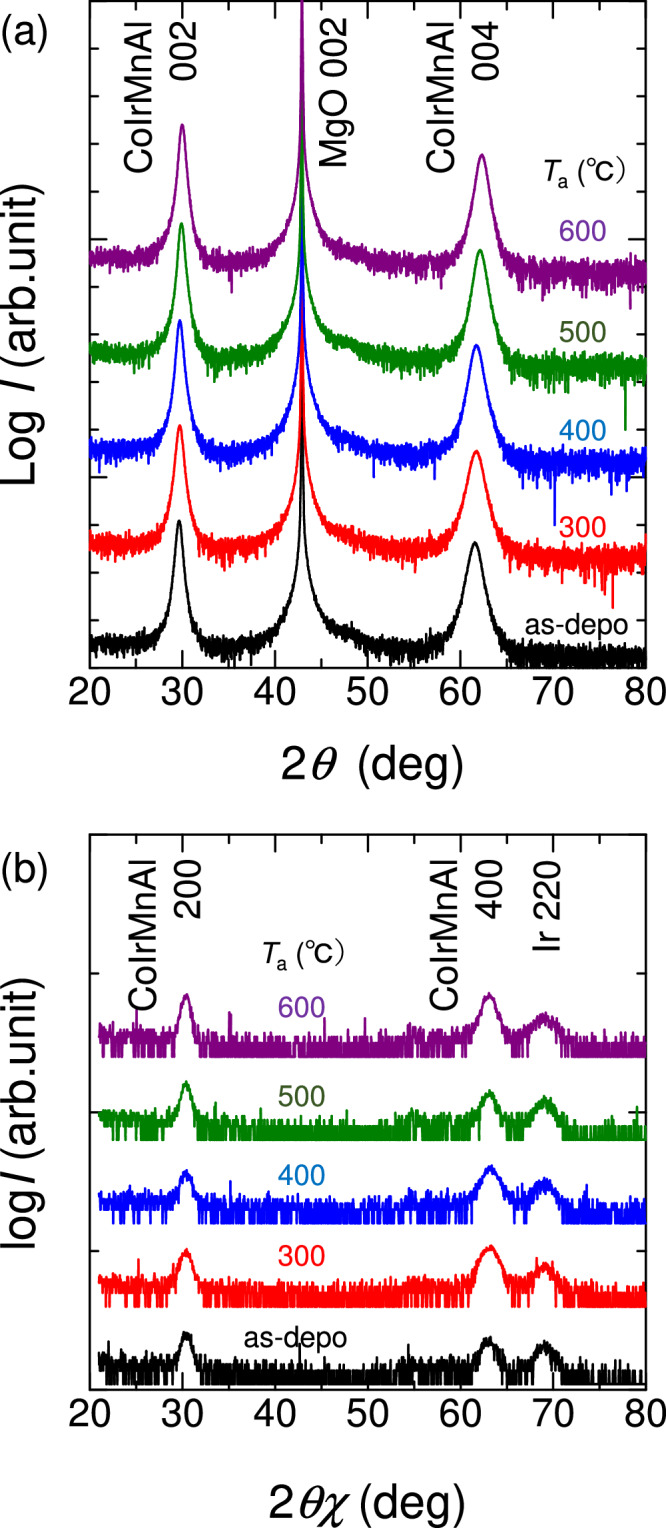
Structural analysis using X-ray diffraction of CoIrMnAl equiatomic quaternary Heusler alloy epitaxial films. **a** Out-of-plane and **b** in-plane XRD patterns for films with various post-annealing temperatures (*T*a). Reprinted with permission from Monma et al.^30^. © 2021 Elsevier.

**Fig. 8:**
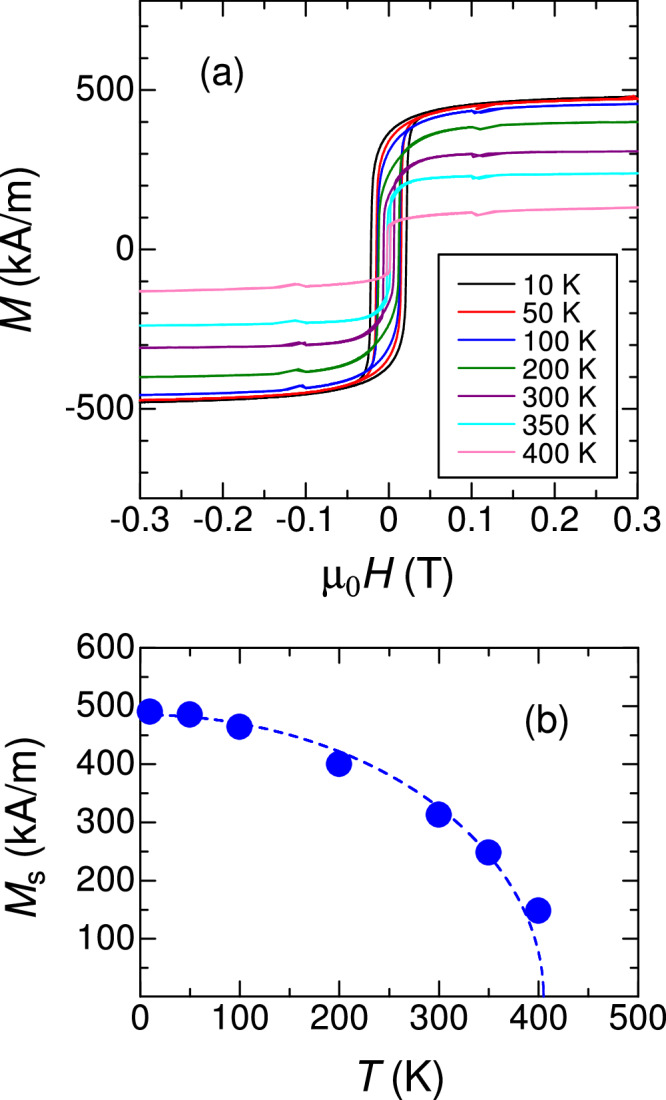
Magnetic properties of CoIrMnAl films. **a** Magnetisation curves and **b** corresponding saturation magnetisation as a function of temperature measured on CoIrMnAl equiatomic quaternary Heusler alloy epitaxial films. Reprinted with permission from Monma et al.^30^. © 2021 Elsevier.

Sample stacking structure of CoIrMnAl was MgO(001) substrate/CoIrMnAl(50 nm)/Ir(3 nm)^30^. CoIrMnAl films were deposited by a co-sputtering technique using Co, Ir, and MnAl alloy targets. The composition of Co : Ir : Mn : Al in the films was evaluated as 28.1 : 27.6 : 20.7 : 23.6 (at%). Out-of-plane XRD indicated the *B*2 ordered (001)-oriented CoIrMnAl films epitaxially grown on MgO(001), as shown in Fig. 7. However, no *L*2_1_ or Y-ordered phase was confirmed via the absence of the superlattice peaks from (111). Magnetisation hysteresis loops were clearly detected as shown in Fig. 8. The saturation magnetisation at 10 K was close to 500 kA/m, which is approximately a factor of 0.7 smaller than the theoretical value, 722 kA/m (4.03 μ_B_/f.u.) in the fully-ordered CoIrMnAl^31^. The Curie temperature was also evaluated as about 400 K, which is also smaller than the values predicted, 584 K, in the fully-ordered CoIrMnAl^31^. It is likely that these discrepancies originate from the site-disorder, as previously discussed^30^.

These studies indicated some advanced techniques of film fabrications may be required for new Heusler alloys in line with the computational prediction of phase stability and preferential site-ordering at ground states. From the theoretical point of view, Y-CoIrMnAl is thermodynamically stable. Thus, more systematic research should be performed, in particular, in bulk. Y-NiCrMnSi is not thermodynamically stable, requiring to obtain this as metastable form. It could be accessed using MBE on an appropriate substrate. Even in sputtering at low deposition temperature, some ordered metastable alloys are obtained using special template. In the case of Mn-based Heusler, we and other group previously discuss template effects promoting film growth with well chemical-ordering even at low temperature^32,33^. To our knowledge to date, the physics behind such special growth process is not yet clear and should be addressed for controlling metastable state of multi-element ordered alloy/compounds.

## Machine learning and first-principles calculations for more complex materials

As observed in the Heusler alloys, atomic disordering plays a critical role in insulating tunnel barrier materials of MTJs. The examples involve spinel-type oxides *AB*_2_O_4_, the particular of which are advantageous in terms of lattice-well-matched heterointerface of bcc-based ferromagnetic materials with a high TMR ratio and/or low resistance-area product (*RA*)^34–37^. The physical properties of TMR junctions desired for spintronic applications can be potentially addressed by wide variations of constituent elements, e.g., their compositions and atomic disordering. To tackle such complexity, data-driven material design has opened a new era of materials science, but materials exploration using conventional machine learning models, such as Bayesian optimisation and Monte Carlo tree search, can make efficient materials exploration difficult due to the combinatorial explosion resulting from the large number of degrees of freedom in the materials themselves.

To address the abovementioned issue, an alternative approach is introduced here, which is not to use only first-principles calculations and machine learning, but to further combine them with quantum annealing (QA) that is inspired from quantum computing technologies recently available. Focusing on atomic disordering of MgGa_2_O_4_ of an MTJ for example, namely Fe/atomic-disordering MgGa_2_O_4_/Fe(001) system, we demonstrate a proof-of-concept study to search for suitable MTJs in terms of energetically stable (lower $\Delta {E}_{{\rm{Total}}}$), higher TMR ratios and lower *RA* figures by optimising an atomic arrangement of Mg and Ga of MgGa_2_O_4_ tunnel barrier.

**Fig. 9:**
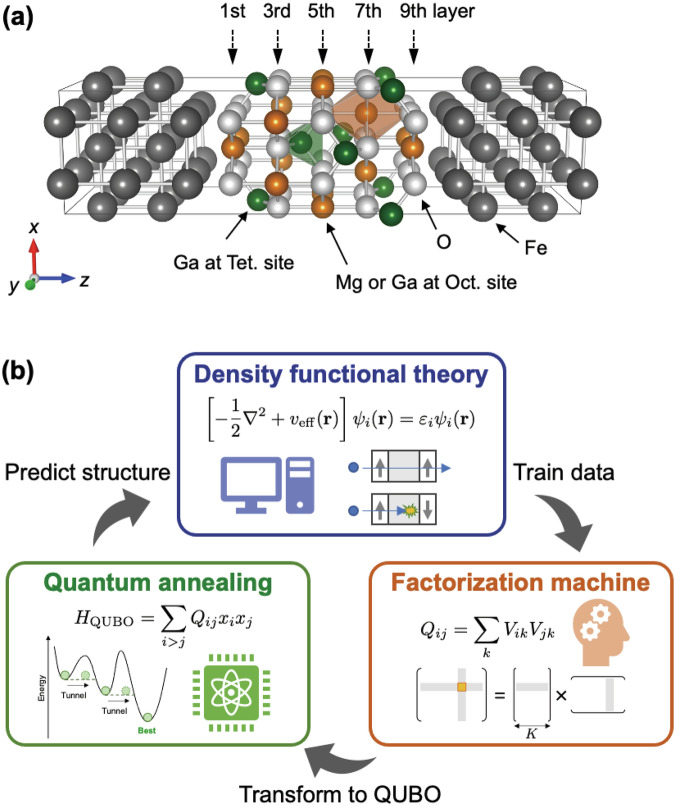
Modelling and calculation processes on MTJs. **a** MTJ structure of Fe/atomic-disordering MgGa_2_O_4_/Fe(001), where Mg and Ga atoms randomly occupy octahedral site (orange site). **b** Frow of calculations combined with QA, FM, and DFT^40^.

In the MTJ with inverse-type spinel MgGa_2_O_4_ as shown in Fig. 9a, all the Mg and half the Ga atoms randomly occupy the octahedral site (orange sites in Fig. 9a) and the remaining Ga atoms occupy the tetrahedral site (green sites in Fig. 9a). In this work, the atomic arrangement of Mg and Ga at the octahedral sites, in which the number of total combinations can be defined as ${10}_{{C}_{5}}= 252$, can be optimised by first-principles calculations of density functional theory (DFT), machine learning and QA techniques. In the QA-based framework, the combinatorial optimisation problem is replaced with an Ising model, and the QA obtains the lowest Ising energy of the given Ising Hamiltonian, which describes well the relationship between physical properties and materials expressed by quantum bits (qubits). An Ising model Hamiltonian can be constructed by using a machine learning, so-called factorization machine (FM)^38^. In the FM model, the target property of interest can be expressed by atomic arrangement of Mg and Ga as follows:

4 $$f({\boldsymbol{x}})=b+\mathop{\sum }\limits_{i=1}^{N}{q}_{i}{x}_{i}+\mathop{\sum }\limits_{i=1}^{N-1}\mathop{\sum }\limits_{j=i+1}^{N}\langle {v}_{i},{v}_{j}\rangle {x}_{i}{x}_{j},$$

where $N$ is the number of qubits corresponding to disordering sites [$N=10$ in this work as shown in Fig. 9a] and a vector ${\boldsymbol{x}}={\boldsymbol{\{}}{x}_{1},\,{x}_{2},\,\ldots ,\,{x}_{N}{\boldsymbol{\}}}$ is a descriptor defining an atomic arrangement of Mg and Ga (${x}_{i}=0$ for Mg and ${x}_{i}=1$ for Ga at disordering site $i$). The fitting model parameters are bias term $b$, linear term ${q}_{i}$ and quadratic term $\langle {v}_{i},{v}_{j}\rangle ={\sum }_{k}^{K}{V}_{ik}{V}_{jk}$. A hyperparameter $K$ determines a rank of FM model and optimised to be 7, 2, and 3 for the optimisation of $\Delta {E}_{{\rm{Total}}}$, TMR, and RA, respectively, in this work. The fitted model parameters, ${q}_{i}$ and $\langle {v}_{i},{v}_{j}\rangle$, can be transformed into the quadratic unconstrained binary optimisation (QUBO) form to solve the Ising model by the QA as below:

5 $$\begin{array}{rcl}{Q}_{ij}=\left\{\begin{array}{l}{q}_{i}\,(i=j)\\ \langle, {v}_{i},{v}_{j}\rangle \,(i\,\ne \,j)\end{array}\right.\end{array}.$$

The Ising Hamiltonian called *H*_QUBO_ is then given as

6 $${H}_{{\rm{Q}}{\rm{U}}{\rm{B}}{\rm{O}}}=\mathop{\sum }\limits_{i < j}{Q}_{ij}{x}_{i}{x}_{j}+\alpha {\left(\mathop{\sum }_{i=1}^{N}{x}_{i}-\frac{N}{2}\right)}^{2}.$$

The first term is a cost function, and the second term is a penalty term to constrain the atomic composition of Mg and Ga to be 1:1. A hyperparameter $\alpha$ (real number, $\alpha > 0$) is to control the strength of penalty term. The QA solves the *H*_QUBO_ and the solution with the lowest Ising energy is the next candidate of Mg/Ga atomic arrangement for which target physical property should be evaluated by the DFT calculations. The total energy, ground-state electronic structures, and spin-transport properties are obtained from first-principles calculations with the basis of linear combination of atomic orbitals, implemented in the Quantum Atomistix Toolkit (Quantum-ATK) simulation^39^. The GGA is employed for the exchange-correlation functional^18^, For the details of calculations method, refer to ref. ^40^. Our calculation flow, FM + QA + DFT, is shown in Fig. 9b. The FM + QA + DFT is referred to FM + QA (DFT is omitted) hereafter.

**Fig. 10:**
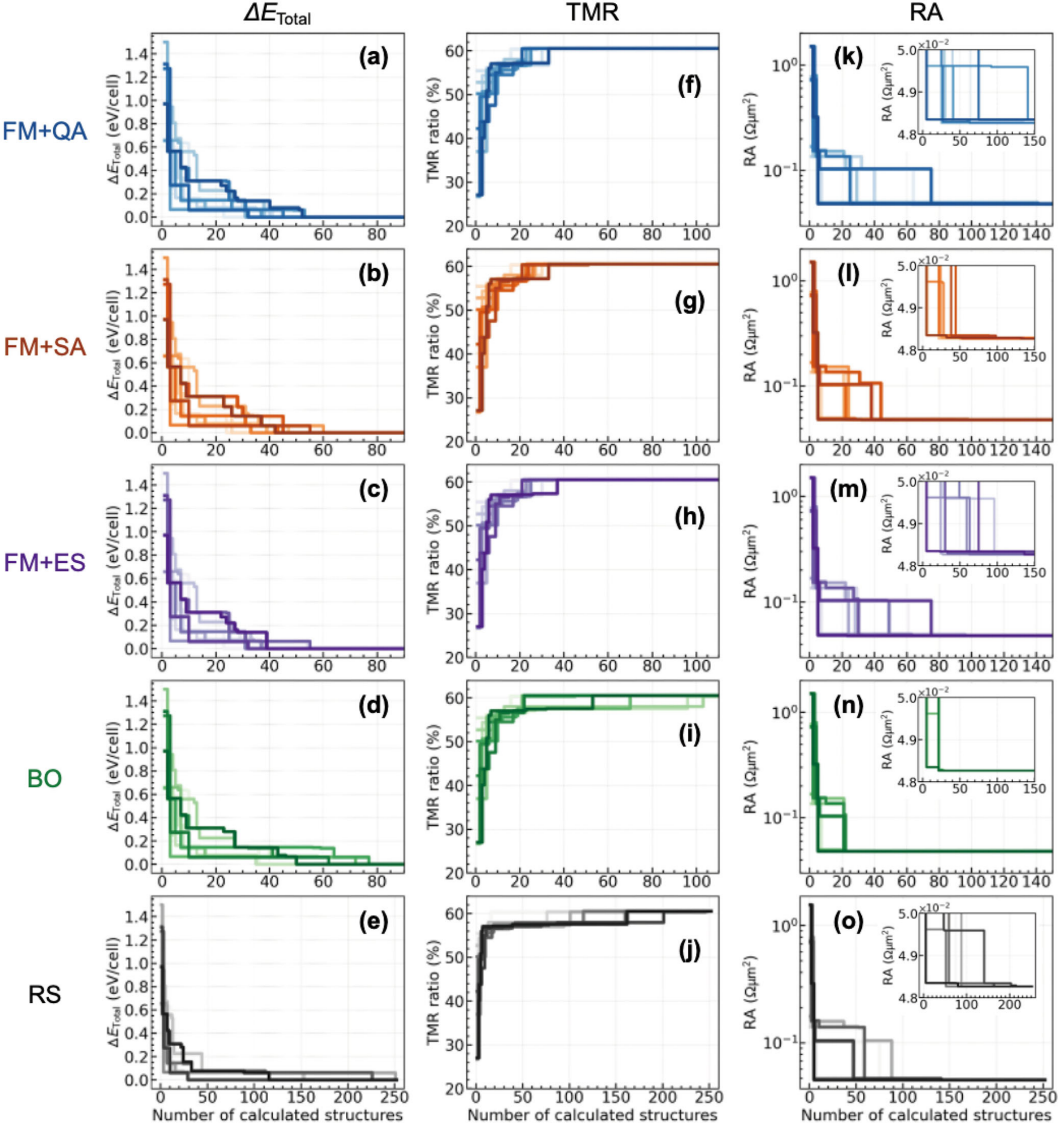
History of the calculated number of structures required to obtain the best MTJ structure. **a**–**e** $\Delta {E}_{{\rm{Total}}}$, (**f**)–(**j**) TMR, and (**k**)–(**o**) RA obtained by FM + QA, FM + SA, FM + ES, BO, and RS, respectively. In each property and method, 10 trials were carried out by using the training dataset including 20 MTJ structures. The horizontal axis includes those initial training dataset, the range of which is different between the RS and others^40^.

In Fig. 10, the results of FM + QA of the searching history to obtain the suitable MTJ are shown for the $\Delta {E}_{{\rm{Total}}}$, TMR, and RA. To investigate an effectiveness of FM + QA, FM + simulated annealing (FM + SA), FM + exact solver (FM + ES) (In the FM+ES, the Ising energy is evaluated for all the atomic arrangements (***x***’s) of the training data, and then the vector x giving the lowest Ising energy is always selected as the next candidate.), Bayesian optimisation (BO), and random search (RS) were also performed, where 10 trials were carried out using different initial training datasets that include randomly selected 20 MTJ structures. For the $\Delta {E}_{{\rm{Total}}}$, as shown in Fig. 10a the FM + QA obtains successfully the suitable MTJ within approximately 50 structures (including the training data of 20 structures), and this is comparable to that of FM + ES (Fig. 10c), verifying our FM + QA method. The efficiency obtained from the FM + QA is slightly faster than the FM + SA (see Fig. 10b) and significantly than the BO and RS (see Fig. 10d, e). For the TMR, similar searching efficiency is obtained among FM + QA, FM + SA, and FM + ES (Fig. 10f–h), and this efficiency is much better than those of BO and RS (see Fig. 10i, j). From these results, thus, the FM + QA might be a promising approach in the search for the preferred MTJ with the energetically stable atomic disordering and the highest TMR property.

On the other hand, there remains a difficulty in optimising the atomic arrangement for MTJs with lower RA by using FM + QA. Figure 10k shows that the variation of the number of structures required to be optimised is very wide, depending on the initial training datasets, with a maximum of over 150 structures required (see the inset). This feature is confirmed even in FM + SA and FM + ES (see Fig. 10m, n). These results seem to reflect the inaccuracy of the FM training, from which the *H*_QUBO_ is constructed, rather than the performance of QA solver. Therefore, it is difficult to reach an optimal solution, i.e., an MTJ with lower *RA*, from such *H*_QUBO_. In contrast, the BO efficiently reaches the best MTJ with the lowest *RA*.

Here potential solutions to improve the robustness of the FM model for *RA* optimisation are discussed. One may obtain better performance of FM model with the hyperparameter *K* greater than *K* = 10, although the *K* is set to 3, yielding the best performance from a pre-test validated within *K* = 1 ~ 10 in this proof-of-concept study. Additionally, the size of database used in this demonstration, *i.e*., 20 training data, may be too small and/or too sparce to adequately train the relationship of the *RA* and atomic arrangement in the FM model. The latter would be more serious in defining the generalisability of FM model, but should be mitigated in a natural way at the practical level of FM + QA( + DFT), where combinatorial explosion occurs, so that the number of training dataset is expected to be larger than that of this demonstration. Furthermore, in a real problem, finding several good material candidates is beneficial for device developments by materials informatics, rather than finding only one best solution. In this context, our demonstration, although still inferior to BO, achieves efficient search performance comparable to those of $\Delta {E}_{{\rm{Total}}}$ and a TMR ratio for *RA* optimisation (see searching efficiency results denoted as “RA*”, in Fig. 4 of ref. ^37^).

To conclude, we propose an alternative approach adaptable to combinatorial explosion problems by combining state-of-the-art QA, FM, and DFT techniques and applied it to optimisation problem of an atomic disordering in MTJs with spinel-oxide tunnel barrier, MgGa_2_O_4_. Our proof-of-concept study achieved higher searching efficiency in particular physical properties compared to the conventional machine learning, such as Bayesian optimisation, while leaving room for improvement of our method. The present approach may offer a breakthrough to overcome the combinatorial explosion, which is inaccessible to conventional machine learnings regardless of whether the system is ordering and disordering. More specifically, the FM + QA + DFT is applicable to multi-component materials such as Heusler alloy families once the combinatorial optimisation problem of atomic elements, compositions, defects, crystal structures, and other degrees of freedom of interest can be mapped onto the Ising model.

## Summary

By using machine learning, we successfully fabricated new Heusler alloy films. Such virtual screening can save time significantly to develop a new spintronic material. Experimentally they show some disordering issues to demonstrate their properties as predicted. This may be caused by one of the following reasons: In the virtual prediction, (i) one should take the corresponding phase diagrams into account, meaning the experiments are correct or (ii) improve the growth process, indicating the calculations are correct. (i) For example, an alloy consisting of Ni, Mn and Si has been reported to contain almost 100 phases^41^, which cannot be considered in the machine-learning prediction and may not be well represented by the total crystalline energy calculated by first principles. Alternatively the quantum annealing can be adopted for further optimisation of the system with complex structures. (ii) In a quaternary Heusler alloy with the form of *XX’YZ*, the *X* (and/or *X’*) and *Y* atoms can be replaced, such as Mn and Ni, differentiating the corresponding crystalline structures. However they cannot be experimentally distinguished with the current technology. We hence need to develop these additional two approaches for more precise prediction and development.

## Data Availability

Data is provided within the manuscript.
